# Teachers’ burnout: A SEM analysis in an Asian context

**DOI:** 10.1016/j.heliyon.2019.e03144

**Published:** 2020-01-09

**Authors:** Lantip Diat Prasojo, Akhmad Habibi, Mohd Faiz Mohd Yaakob, Robin Pratama, Mat Rahimi Yusof, Amirul Mukminin, Suyanto, Farida Hanum

**Affiliations:** a Universitas Negeri Yogyakarta, Indonesia; b Universitas Jambi, Indonesia; c Universiti Utara Malaysia, Malaysia

**Keywords:** Teachers, TSC, TE, Burnout, Gender, Teaching experience, Education, Educational psychology, Health education, Pedagogy, Teaching research, Psychology

## Abstract

Researchers in educational psychology have researched Teacher Self-Concept (TSC) and Teacher Efficacy (TE) as two main predictors predicting burnout. Guided by a model developed by Zhu, Liu, Fu, Yang, Zhang & Shi (2018), the researchers aimed at building a model involving TSC, TE, and three components of burnout; Emotional Exhaustion (EE), Depersonalization (DP), and Reduced Personal Accomplishment (RPA) through Structural Equation Modeling (SEM). The researchers investigated predicting factors of burnout by reporting TSC and TE that might directly affect the components and examine the probability of TE to become a mediator of the correlation between TSC and burnout. This research also examined whether the difference emerges constantly among demographic information (gender and teaching experience) regarding all involved variables. A sample of 876 teachers across three Indonesian provinces completed a printed form of questionnaires. Some statistical procedures namely Content Validity Index (CVI), Exploratory Factor Analysis (EFA), Confirmatory Factor Analysis (CFA), Covariance-Based Structural Equation Modeling (CB-SEM), and t-test were conducted. Findings informed that the model is valid and reliable. TSC could directly affect EE, DE, and RPA, as well as indirectly influence them mediated by TE. Besides, TE is also reported to have significant relationships with EE, DE, and RPA. No significant differences in terms of age and teaching experiences emerge, except for EE.

## 1 Introduction

The teaching profession has been considered as a highly stressful job with multiple stressors resulting in a high risk of burnout (Friedman, 2003). Work-related pressures like catching up with submission deadlines, adapting to unhealthy working conditions, and dealing with problematic students were among the challenges faced by the teachers. The constant exposures of the teachers on these situations, if not properly handled, may turn them into possibilities of experiencing a malfunctioned reaction called “burnout”.

Theoretically, there are three components of burnout that have been introduced and conceptualized called “Maslach Burnout Inventory” (MBI) (Maslach et al., 1996). The parts of burnout have gained international acceptance and were validated in many studies (Chan, 2007) while investigating the specific predictors on burnout components is an exciting research topic. Teacher's self-perceptions have been now significantly and increasingly investigated as essential factors affecting burnout. Guided by the model established by Zhu et al. (2018) in China and the model of TE by Tschannen-Moran et al. (1998), this research aimed at investigating whether and how TSC and TE can affect the components of burnout speculating that TSC would affect burnout through TE. Besides, the researchers also examined the significant differences in term of gender and teaching experiences for all constructs.

## 2 Literature review

### 2.1 Teacher burnout

Maslach (1999) defined “burnout” for a malfunctioned reaction to severe psychological and relational stressors, especially at the workplace. Moreover, it happens even in the interpersonal-purposed occupation, including teaching professions. Workers who have severe burnout syndrome influence their overload to their peers as well as their own experience. They might perform a syndrome of EE, DE, RPA (Maslach, 1999). In a specific way, EE shows depletion of emotional sources. DE is a detached behavior towards the receivers. RPA refers to a feeling of being incompetent doing a job. These three components have been agreed by many educational academics to be separately investigated constructs as they are influenced by various kinds of cycles (Skaalvik and Skaalvik, 2010) which are also applied in this research context. The burnout model found by Maslach and Leiter (1997) was implemented to elaborate the predictors of teachers’ burnout for Asian context, especially in Indonesia.

### 2.2 Teacher efficacy

Many researchers have explored TE (e.g. Bandura, 1977; Love et al., 2019; Rotter, 1966). In the beginning, Rotter (1966) explored TE from the perspective of teaching activities informing that reinforcement is considered to be not of teachers' control when they perceived that the effects of the environment are more significant than teachers' control or it outreaches their abilities or locus of control. Afterwards, Bandura (1977) stated that efficacy is a belief to achieve goals from one's capabilities namely self-efficacy as future-oriented thought; she/he can decide what activities to choose and how much effort to do as well as how long to deal with emerged challenges. This research adapted Bandura's proposal where TE is a definition of a judgment of one's capabilities to include desired outcomes of student engagement.

The correlational studies between TE and burnout have been investigated by many researchers (e.g. Dicke et al., 2014; Friedman, 2003; Leiter, 1992; Tang et al., 2001; Wang et al., 2015). For example, Leiter (1992) reported TE as an efficacy crisis or Friedman (2003) as a breakdown of efficacy. On the other hand, TE was reported to be positive as a factor protecting someone from burnout or a personal resource to decrease the level of burnout (Dicke et al., 2014; Schwarzer et al., 2000). Quantitatively, Brown (2012) reported that there was a strong relationship between TE to EE and RPA and the relationship between TE and DE. In addition, multivariate analysis for 16 works of literature presented a weak correlation between classroom management efficacy and burnout. Meanwhile, the strongest correlation emerged between personal accomplishment and classroom management efficacy.

### 2.3 Teacher Self-Concept

TSC was defined as teachers’ perception of the evaluation of the effectiveness of instructional activities (Yeung et al., 2014). Teachers with negative TSC can have an emotional problem or stress (Villa and Calvete, 2001), which might cause burnout. Some empirical researchers have investigated the correlation between TSC and burnout (e.g. Friedman and Farber, 1992; Rad and Nasir, 2010; Villa and Calvete, 2001). Friedman and Farber (1992) through a sample of primary school teachers responding to a TSC scale for items like personal competence and burnout revealed that teachers with negative satisfaction of professionalism had a higher chance of suffering burnout. Meanwhile, Rad and Nasir (2010) reported a negative link between TSC and burnout through a sample of 150 senior teachers. Similarly, Villa and Calvete (2001) reported a poor relationship between TSC and burnout.

In this research, TSC is defined as teaching competence beliefs that is different from SE in several terms used in previous studies (Bong and Skaalvik, 2003). Specifically, TSC is a general perspective that uses more general measures. It has significant effects on evaluation which strongly depends on social comparison gaining reflection to perceived competence. On the other hand, SE in this study is examined by a context-based evaluation that focuses on whether capability reaches objective goals or standards.

### 2.4 Mediation effects

Tschannen-Moran et al. (1998), who submitted a model for the natural cycles of TE emphasized the significant role of the cognitive process. Teachers are suggested to assess the resources of efficacy in establishing their TE by analyzing their daily teaching skills and ability as well as instructional tasks. Therefore, the efficacy formation is preceded by the assessment of teaching competence. Poulou (2007) encouraged the theory by investigating the predictors that affect TE. The results informed that self-perception of instructional competence and personal signs as well as positively affected TE. Poulou (2007) opined on the broader construct consisting of general aspects of competence which might have a substantial contribution to TE. Based on these backgrounds, the investigation was carried out to confirm whether TSC has a contribution to burnout through TE.

The researchers further investigated if the mediation emerges continually among demographic information of gender and teaching experience. Gender has been reported to have a significant difference in predicting TSC, TE and burnout. Some studies (Grayson and Alvarez, 2008; Purvanova and Muros, 2010) informed that females showed more EE, while men suffered more DE and RPA. Meanwhile, for teaching experience, young teachers had a more burnout than that of senior ones (Gavish and Friedman, 2010) and more experienced teachers had higher self-efficacy that new teachers did (Tschannen-Moran and Hoy, 2007).

### 2.5 Hypotheses

**Figure 1:**
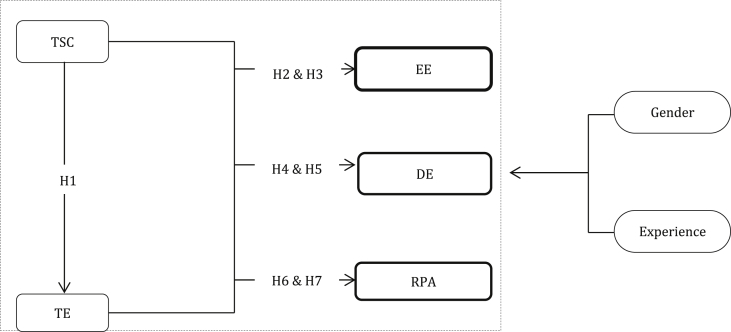
Model of the research.

Six hypotheses were included to help guide the readers of this study. Figure 1 exhibits the proposed path model of the study with six hypotheses.

H1 There will be a significant relationship between TSC and TE

H2 There will be a significant relationship between TSC and EE

H3 There will be a significant relationship between TE and EE

H4 There will be a significant relationship between TSC and DE

H5 There will be a significant relationship between TE and DE

H6 There will be a significant relationship between TSC and RPA

H7 There will be a significant relationship between TE and RPA

## 3 Method

Some procedures were conducted to achieve the purpose of this research, namely; Content Validity Index (CVI), EFA, CFA, CB-SEM, and t-test. Universitas Negeri Yogyakarta that funds this research did not require any ethical approval for the study. Informed consent was obtained from all individual participants involved in the study. The researchers ensured the anonymity of the participants.

### 3.1 Scale development

Five items of TSC were constructed from the TSC Evaluation Scale by Villa and Calvete (2001) measuring teachers’ general self-evaluation of competence. A survey instrument by Yu et al. (1995) was adapted for TE with five items. High scores indicated higher TSC and TE. MBI items (Maslach et al., 1996) were adapted for teacher burnout, comprising three sub-scales: EE (5 items), DE (3 items), and RPA (7 items). The items were measured on a 5-point Likert scale ranging from 1 = never to 5 = always. The adaptation of the instrument involved a discussion with five educational experts through content validity. Therefore, the final decision of eliminating few original items and changing the scales from a 7-point to a 5-point Likert was taken based on the careful consideration and discussion with the experts for context specific instrument (Hair et al., 2010). The researchers also perform CVI, EFA, and CFA to further purify the instrument to fit the context. High scores on the scales specified the high possibility of being burnout. Through back-translation strategies, the translation of the instruments was done from English to Indonesian and Indonesian to English involving two professional translators (Colina et al., 2017).

### 3.2 Content Validity Index (CVI)

A CVI procedure (Lynn, 1986; Habibi et al., 2020; Halek et al., 2017) involving 10 Indonesian educational experts was conducted to establish the validity of the instruments to fit Indonesian educational context. The items were rated on a 4-point scale in the CVI process (1 = not relevant/not clear to 4 = very relevant/very clear) (Halek et al., 2017). The CVI was evaluated for the item level (I-CVI) and scale level (S-CVI). The I-CVI was evaluated by informing a score of 3 or 4 to the experts divided by the experts’ total number (Lynn, 1986). With ten experts, the I-CVI should not be less than the value of .780 (Habibi et al., 2020; Halek et al., 2017). The S-CVI was the average portion of the items on one scale rated 3 or 4 (average agreement by experts = S-CVI/AVE); the acceptable value for the S-CVI is .800 (Halek et al., 2017). Only one item was deleted which scored lower than the I-CVI and S-CVI threshold.

### 3.3 Pilot study

After the CVI process, a pilot study was done with 50 Indonesian teachers from one province. The researchers permitted the teachers to write comments about how they comprehended each item and confusing items were revised. In addition, a reliability test was conducted at this stage by assessing Cronbach's alpha values. No values were found to be less than .700 (Pallant, 2016) as the cut off point for the Cronbach alpha evaluation.

### 3.4 Data collection

As the reliability assessment of the pilot study was done, we distributed the instruments to the participants. Through, simple random sampling, the researchers distributed printed instrument to 1000 Indonesian high school teachers from 3 cities, Jambi, Bandung, and Yogyakarta. Eight hundred and seventy-six data were fully completed and measurable after the data screening process (Table 1). There were 618 (70.54%) female respondents and 258 (29.35%) were males. Their teaching experience varied, 291 (33.22%) teachers whose teaching experience was less than five years and 585 (66.78) whose teaching experience was more than five years. The researchers referred to the theory of Ghaith and Shaaban (1999) who categorized teachers with less than five years teaching experience as novice teachers and with more than five years as experienced teachers.

**Table 1:** Summary statistics of demographic variables.

Variables	N	%
Gender		
Male	618	70.5
Female	258	29.35
Teaching experience		
Less than five years	291	33.22
Five years and more	585	66.78

## 4 Results

Before the main procedures of data analysis (EFA, CFA, and CB-SEM) were conducted, the normality assessment was done by measuring the Skewness, Kurtosis, and Histogram. Hair at al. (2010) recommended the threshold value from -1 to +1 for the Skewness and -2 to +2 for the Kurtosis. Multicollinearity issue happens if the correlation matrix with correlations was more than 0.90 (Hair et al., 2010).

### 4.1 Preliminary analysis

The findings of the Skewness and Kurtosis values of each construct were reported to be satisfactory (Hair et al., 2010). The Skewness values were from -.675 to .004 and the Kurtosis values ranged from -.1.518 to .121. Using histogram that is a graph performing the real form the data distribution shape, the data were found to be normally distributed because they performed a higher distribution in the middle than the edge sides. In terms of multicollinearity, inter-correlations amongst the constructs ranged from .322 to .656. Therefore, the discriminant validities of the variables were reached because the correlation matrix with correlations was less than 0.90 (Hair et al., 2010). The average mean value of TSC is (M = 3.75; SD = .492); TE (M = 4.05; SD = .580), EE (M = 3.82; SD = .662), DE (M = 3.78; SD = .556), RPA (M = 3.90; SD = 626). Overall, the summary statistics of each item is informed in Table 2.

**Table 2:** Summary statistics of study constructs.

Scale items	M (SD)	SD
MBI		
EE-Item 1	3.81	.758
EE-Item 2	3.72	.847
EE-Item 3	3.89	.833
EE-Item 4	3.67	.789
EE-Item 5	4.01	.810
DE-Item 1	3.96	.672
DE-Item 2	3.54	.672
DE-Item 3	3.83	.694
RPA-Item 1	3.94	.835
RPA-Item 2	3.96	.842
RPA-Item 3	3.89	.745
RPA-Item 4	3.84	.782
RPA-Item 5	3.84	.794
TSC		
TSC-Item 1	4.08	.706
TSC-Item 2	4.03	.699
TSC-Item 3	4.12	.707
TSC-Item 4	4.11	.687
TSC-Item 5	3.90	.750
TE		
TE-Item 1	3.69	.698
TE-Item 2	3.80	.611
TE-Item 3	3.74	.626
TE-Item 4	3.67	.674
TE-Item 5	3.81	.654

### 4.2 Reliability and EFA results

The main examination of the data was conducted through Cronbach's alpha to see the reliability, EFA, CFA for the factor analysis assessment, and CB-SEM for deciding the fit model (Hair et al., 2010). Since the adapted instrument was translated form English into Indonesian, EFA was conducted to obtain new constructs from the study sample characteristics (Hair et al., 2010). For the EFA procedure, component principal analysis (PCA) approach was used to formulate uncorrelated linear combination against observable variables. For the EFA, Kaiser Meyer Olkin (KMO), Bartlett's Test of Sphericity, eigenvalue, communality, and factor extraction were computed. The KMO value, greater than .500, is regarded as appropriate while the value of >.800 is considered to be highly satisfactory, and Bartlett's Test of Sphericity is significant at p < .050. Factors, with an eigenvalue <1.0, should be deleted from the factor list while communality value should not drop for less than .300 (Hair et al., 2010). The value of factor loading for each item (>.400) is significant to confirm the questionnaire (Hair et al., 2010).

The complete explanation of reliability and EFA procedures can be seen in Table 3. The KMO value of the data was highly satisfactory (.925) and Bartlett's Test of Sphericity was 10084.447 (significant at *p* < .001). Using Varimax rotation, five factors were achieved with eigenvalues from 1.014 to 8.975. No issue emerged for the Communality. One item (EE5) was eliminated due to highly detected cross-loading. The Cronbach's alpha (α) values ranged from .754 to 875.

**Table 3:** EFA results and Inter-item reliability test.

Factor		1	2	3	4	5	Communality	Eigenvalue	α
TSC	TSC3	.793					.639	8.975	.875
	TSC1	.780					.565		
	TSC4	.771					.570		
	TSC2	.732					.531		
	TSC5	.533					.597		
RPA	RPA2		.766				.707	1.722	.842
	RPA3		.758				.643		
	RPA1		.712				.744		
	RPA4		.709				.769		
	TRA5		.561				.515		
TE	TE1			.712			.624	1.449	.810
	TE3			.668			.760		
	TE5			.660			.675		
	TE4			.653			.717		
	TE2			.592			.667		
EE	EE2				.774		.745	1.213	.859
	EE4				.741		.633		
	EE3				.635		.713		
	EE1				.610		.768		
DE	DE2					.827	.683	1.014	.754
	DE1					.742	.620		
	DE3					.709	.490		

### 4.3 Testing measurement model

Following the EFA procedure, CFA was examined using three indices to confirm the EFA: the Chi-Square Test (χ2), the Root Mean Square Error of Approximation (RMSEA), and the Standardized Root Mean Square of Residual (SRMR) were all measurements of this procedure. The relative goodness-of-fit indices recommended for this study are; Comperative Fit Index (CFI) and Tucker-Lewis Index (TLI). To achieve a fit model, the cut-off points were implemented for each measurement; RMSEA = ≤ .080 (Kelley and Lai, 2011), SRMR = ≤ .08 (Hu and Bentler, 1999), TLI = ≥ .900, and CFI = ≥ .900 (Hair et al., 2010; Kline, 2005).

Using different samples, CFA was conducted in order to verify the EFA result or test measurement model. CFA can facilitate further evaluation regarding the fitness of the model in line with the structure of the factors (Hair et al., 2010). The initial measurement model did not achieve the fit model; RMSEA = .069, SRMR = .026, TLI = ≥ .876, and the CFI = .918. In this initial measurement TLI did not meet the cut-off point. Besides, two items (RPA5 & TE4) gained low loading values (Hair et al., 2010). Therefore the two items were eliminated. All loading factors after the elimination of the two items were appropriate that ranged from .621 to .896 (Table 4). The final results show good fit indices for the CFA; χ2 = 830.366, χ2/df = 5.190, RMSEA = .069, SRMR = .025, TLI = ≥ .914, and the CFI = .928. Cronbach's alpha (α) coefficients, composite reliability (CR) and average variance extracted (AVE) were calculated to determine the reliability of the instrument. Hair et al. (2010) and Pallant (2016) indicated that alpha values of 0.60–0.70 are satisfactory. CR should be more than 0.60, whereas AVE should be above 0.50. Table 4 performs that all values of α, CR, and AVE exceed the requirements.

**Table 4:** CFA results of Final measurement model (all construct).

Construct	Item	Standardized loadings	(CR)	(AVE)	α
Emotional Exhaustion	EE1	.742	.778	.860	.859
	EE2	.793			
	EE3	.797			
	EE4	.780			
Depersonalization	DE1	.682	.709	.753	.754
	DE2	.682			
	DE3	.763			
Reduced Personal Achievement	RPA4	.621	.772	.859	.842
	RPA3	.706			
	RPA2	.896			
	RPA1	.865			
Teacher self-concept	TSC1	.707	.698	.792	.828
	TSC2	.717			
	TSC3	.663			
	TSC5	.706			
Teacher efficacy	TE1	.753	.745	.834	.792
	TE2	.758			
	TE3	.810			
	TE5	.662			

CR: composite reliability; AVE: Average Variance Extracted; α: Crobach's alpha.

### 4.4 Relationship between TSC, TE and burnout

To support the path analysis of CB-SEM, the correlational analysis was conducted through Pearson correlation coefficient. This study implemented general *r* value guideline (Evans, 1996); 00-.19 as “very weak”; .20-.39 as “weak”; .40-.59 as “moderate”; .60-.79 as “strong”, and .80–1.0 “very strong”. The findings of the study (Table 5) indicated that the correlation between TE and TSC is significant and moderate (*r* = .564, *p* < .01), TE and EE is significant and strong (*r* = .657, *p* < .01), TE and DE is significant and moderate (*r* = .452, *p* < .01), TE and RPA is significant and moderate (*r* = .550, *p* < .01), TSC and EE is significant and strong (*r* = .650, *p* < .01), TSC and DE is significant and moderate (*r* = .440, *p* < .01), TSC and RPA is significant and moderate (*r* = .578, *p* < .01), EE and DE is significant and weak (*r* = .380, *p* < .01), EE and RPA is significant and moderate (*r* = .656, *p* < .01), DE and RPA is significant and weak (*r* = .332, *p* < .01).

**Table 5:** Pearson correlation results among the constructs (** *p* < .01).

	TSC	EE	DE	RPA
TE	.564**	.657**	.452**	.550**
TSC	1	.650**	.440**	.578**
EE		1	.380**	.656**
DE			1	.322**
RPA				1

Similar to the testing measurement model, some threshold points were also implemented for each measurement to assess the final model; RMSEA = ≤ .080, SRMR = ≤ .08, TLI = ≥ .900, and the CFI = ≥ .900. The findings of the SEM report the structural model of χ2 = 881.682, χ2/df = 5.4090, RMSEA = 0.071, TLI = .910 and CFI = .923. All loading values were in the range from .62 to .90, exceeding the desirable standard of .50 (Hair et al., 2010). The hypothetical structural information for the CFA became the finalized model indicating correlation among TSC, TE, EE, DE, and TRA for the context of Indonesia or other developing countries. The final model from this research can be used as an option to support or argue the previous research on factors affecting burnout among teachers. In conclusion, the evaluations of the modeling were fit for the Indonesian context.

**Figure 2:**
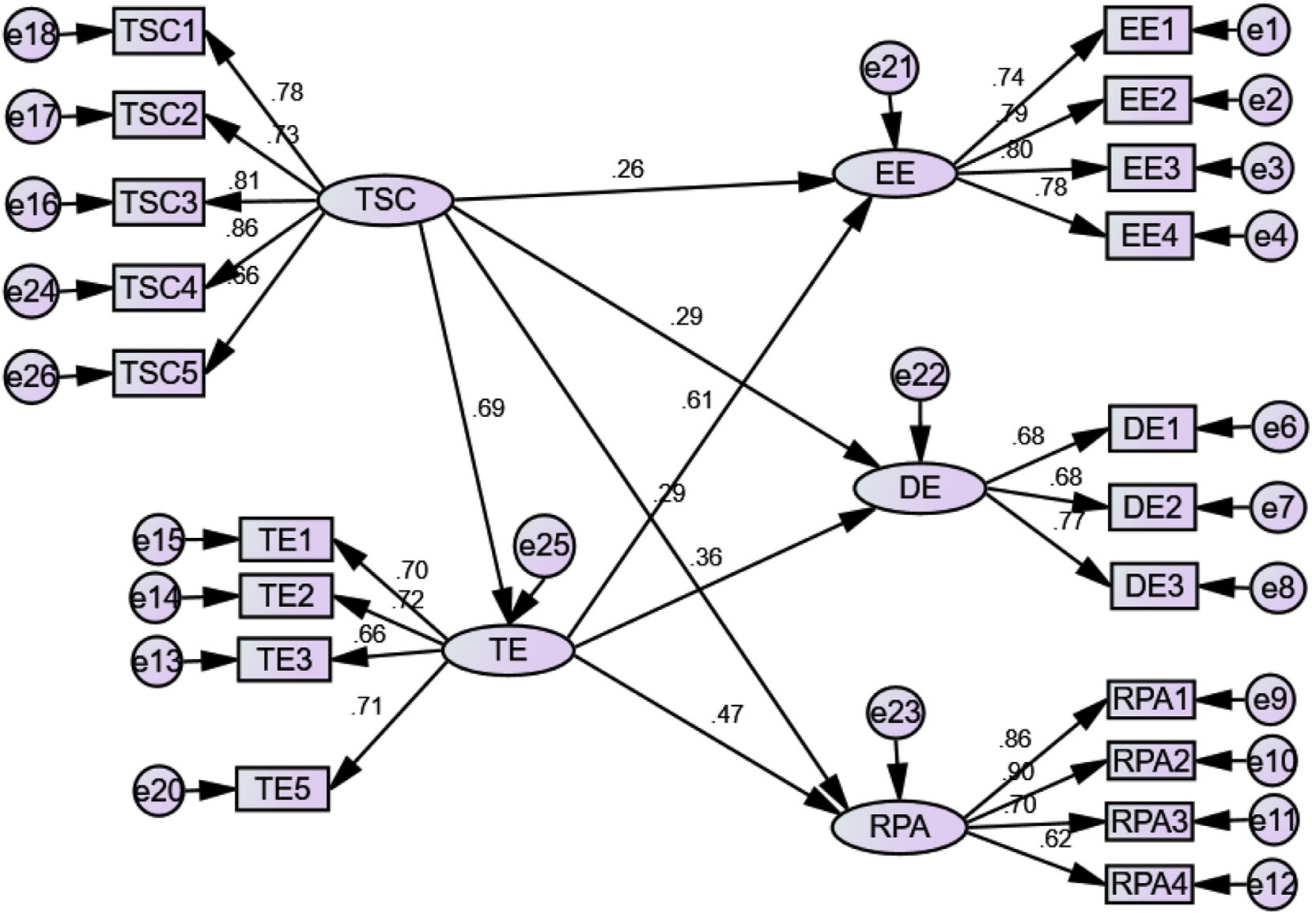
Final study.

TSC was included as an independent variable that predicted EE, DE, and RPA as burnout components, and TE was included as variable mediating TSC for burnout in the modeling process for this research. TSC had the stronger effect to EE (β = .69; *p* < .001) than DE (β = .61; *p* < .001) and RPA (β = .29 *p* < .001). TE was correlated to all three burnout components. The TE effect of mediation could be essential. Through the mediation of TE, TSC had a greater indirect effect on DE (β = .29; *p* < .001) and RPA (β = .29; *p* < .001) than that of EE (β = .26; *p* < .001) indicating a partial mediation effect. Additionally, the final result of the SEM process is depicted in Figure 2 and Table 6.

**Table 6:** SEM results for the structural model.

Hypothesis	Path	β	S.E.	C.R.	p	Label
H1	TSC→TE	.69	.053	15.496	<.001	Yes
H2	TSC→EE	.26	.063	11.168	<.001	Yes
H3	TSC→DE	.29	.057	5.860	<.001	Yes
H4	TSC→RPA	.29	.077	9.128	<.001	Yes
H5	TE→EE	.61	.044	5.795	<.001	Yes
H6	TE→DE	.36	.046	5.002	<.001	Yes
H7	TE→RPA	.47	.060	6.209	<.001	Yes

### 4.5 Significance differences

The study also investigated whether the demographic information (gender and teaching experience) differs regarding all constructs (TSC, TE, EE, DE, RPA). The t-test results reported that there is no statistical difference between male and female teachers concerning all constructs. Similarly, no significant differences emerge for teaching experience but one construct, DE (*t* = -3.417; *p* < .005) Complete information and comparison of the values across the demographic can be seen in Table 7.

**Table 7:** Differences regarding gender and teaching experience.

Construct	Demographic information	n.	Mean	SD	t	df	p

TSC	Female	618	4.08	.619	-.781	874	.435
	Male	258	4.11	.540			
TE	Female	618	3.74	.517	-1.039	874	.299
	Male	258	3.79	.485			
EE	Female	618	3.76	.674	-.432	874	.666
	Male	258	3.79	.682			
DE	Female	618	3.77	.569	-1.027	874	.305
	Male	258	3.81	.522			
RPA	Female	618	3.91	.664	-.064	874	.949
	Male	258	3.91	.687			
TSC	Beginner	291	4.04	.578	-1.301	874	.194
	Experienced	585	4.10	.605			
TE	Beginner	291	3.73	.468	-1.233	874	.218
	Experienced	585	3.77	.526			
EE	Beginner	291	3.72	.661	-1.323	874	.186
	Experienced	585	3.79	.684			
DE	Beginner	291	3.68	.543	-3.417	874	<.005
	Experienced	585	3.82	.557			
RPA	Beginner	291	3.87	.603	-.892	874	.373
	Experienced	291	4.04	.578			

## 5 Discussion

Through CVI, reliability assessment after the pilot study, and factor analysis (EFA and CFA), the model that involves TSC, TE, and burnout components (DD, DE, and TRA) is informed to be valid and reliable for (Hair et al., 2010). Through some modifications and deletions of few items, the model is decided to fit the relationship between predictors and teachers’ burnout in the context of Asia, especially Indonesia with all values measuring the model fulfill the common threshold values used by SEM researchers (Pallant, 2016).

The researchers examined TSC and TE as the exploring factors to predict teachers' burnout. The study includes a large sample of Asian culture and tested whether teachers’ burnout varies based on their demographic information. The findings are hoped to facilitate a current understanding of the systematic function of TSC and TE in predicting EE, DE, and TRA as the burnout components in a different context and circumstance. Based on CB-SEM analysis, the findings informed that TSC directly affects burnout, as well as indirectly influences burnout via TE. The findings of the research facilitate our hypotheses that have been assumed based on the study reported by Tschannen-Moran et al. (1998) and Zhu et al. (2018). Generally, TSC provided a basis offer for teachers to perform judgment on efficacy. TSC and TE could work together as affecting protective factor to variables of burnout. The direct affecting findings of this are not accordance with previous research (e.g. Rad and Nasir, 2010; Villa and Calvete, 2001) that informed the negative correlation between TSC and all burnout variables. However, Zhu et al. (2018) disclosed that TSC was confirmed to positively influence TRA. Compared to TSC, TE had better influences on burnout components, EE, DE, RPA.

TSC has been described as a general belief of competence in instruction. This general belief can directly affect all components of burnout, TE, DE, and RPA and indirectly impact the other two syndromes of burnout through TE. Compared to TSC, TE had less impact on burnout. A possible elaboration for this might be that TE reflects a more specific perception of competence; thus, low TE could cause the increasing of burnout. Even though this study was carried out with teachers from a non-western psychosocial circumstance, teachers' professional appreciation has also been conducted based on individual achievement, similar to teaching appreciation in many western countries. Therefore, a social comparison should always be conducted in research about teachers’ burnout in the future with different cultures of the samples (Zhu et al., 2018).

The findings of the also informed that most demographic information (gender and teaching experience) do not significantly differ regarding all constructs (TSC, TE, EE, DE, RPA). Only one construct in the burnout component (DE) is found to be different regarding teaching experience. The results of current study argue what Grayson and Alvarez (2008) and Purvanova and Muros (2010) found in the previous studies that informed that female respondents showed more EE, while men had more DE and RPA. On the other hand, for teaching experience, teachers with few experiences had more burnout than teachers with many experiences (Gavish and Friedman, 2010) and more experienced teachers had higher TE than new teachers did (Tschannen-Moran and Hoy, 2007). Even though, the differences are reported to be insignificant in terms of teaching experience; it is suggested to maximally help teachers decrease their feeling of burnout by providing them with more teaching training to improve their skills and experience.

## 6 Conclusion and recommendation

All predictors (TSC and TE) have been reported to be significant in predicting teachers' burnout. The findings of the research advocate the proposed model. The model can be a guidance for future Asian researchers to predict teachers’ burnout. On the other hand, the study informed that gender and teaching experience are not significantly different for all variables; one construct is reported to be different regarding teaching experience for DE. Based on the results of this study, the needs of building TSC and TE to prevent burnout is recommended for the Asian context. Educational stakeholders should ensure that schools can be institutions that teachers will be successful in promoting learning activities. As a result, TSC and TE are fostered and burnout can be eliminated. The simplest and most practical way for school is to facilitate teachers multiple opportunities experiencing beneficial teaching such an open course with some motivated students. Through this way, they can be motivated to keep developing their own style and influence other teachers for learning to teach. Working with peers would also be useful while making lesson plans. With this kind of self-learning and peer learning, they can accumulate experience and verbal encouragement and appreciation from expert teachers that will improve TSC and TE (Tschannen-Moran et al., 1998).

Some advantages of the concept and measure of this study can be detected. TSE was a concept of a general belief of teaching competence. The sample represented an appropriate size. The shifting condition in Indonesia from achievement attainment to process-based instruction in the 21^st^-century education make research about teachers' burnout, to be a priority since students’ academic performance is now less valued causing the students to have more problems with behaviors or discipline problems. Therefore, this research considered future research motivating students to both attain achievement and decrease student discipline problems. A limitation that should be considered is that the adopted TE items focused on promoting students attain good achievements, not their discipline problems. Since there have been many reports with students in the context of Indonesia, a more measurement to deal with students discipline problems must be considered. Another lack of this research is the method of this study; in-depth and enriched information through qualitative inquiry is recommended for future researchers, especially in developing countries.

## Declarations

### Author contribution statement

Lantip Diat Prasojo: Conceived and designed the experiments; Performed the experiments; Wrote the paper.

Akhmad Habibi: Conceived and designed the experiments; Analyzed and interpreted the data; Contributed reagents, materials, analysis tools or data; Wrote the paper.

Mohd Faiz Mohd Yaakob, Mat Rahimi Yusof, Amirul Mukminin, Suyanto: Contributed reagents, materials, analysis tools or data; Wrote the paper.

Robin Pratama: Performed the experiments; Analyzed and interpreted the data; Contributed reagents, materials, analysis tools or data.

Farida Hanum: Analyzed and interpreted the data; Wrote the paper.

### Funding statement

This research was fully funded by Universitas Negeri Yogyakarta No. 4868AA/UN34/SPK/2019 done from January to June 2019.

### Competing interest statement

The authors declare no conflict of interest.

### Additional information

No additional information is available for this paper.
